# Get up and go! An Evaluation of Fitness and Memory

**DOI:** 10.1093/geroni/igab046.2881

**Published:** 2021-12-17

**Authors:** Christina Nunez, Alexandria Nuccio, Sophia Perez, Charles Golden

**Affiliations:** Nova Southeastern University, Fort Lauderdale, Florida, United States

## Abstract

As we age, exercise is increasingly important for physical health and well-being. Recent studies have shown that exercise is associated with cognitive performance across multiple domains, specifically memory, a common complaint for older adults. Data included a ten-word list of delayed recall, a clock drawing activity, and a sit-to-stand task (i.e., a low impact sub-maximal test of functional fitness) derived from the National Health & Aging Trends Study Database (NHATS Round 9). A total of 4977 participants were included in the analysis which was predominantly white (69.7%), non-Hispanic (94.5%), female (59.2%), and between the ages of 70-84 (62.7%). A hierarchical linear regression revealed that performance on the sit-to-stand task positively predicted performance on delayed recall, F(4,3914)=245.141, p<.001, and on the clock drawing activity, a common screening task for cognitive decline, F(4,2893)=115.470, p<.001; accounting for 20.1% and 10.6% of the variability, respectively, over and above known demographic variables. These findings indicate that exercise may be one of many factors that is associated with memory and cognitive decline. Given the continuation of quarantine procedures, these findings come at a time of significant clinical relevance. Research shows that many individuals slowed down because of the COVID-19 pandemic, and current findings suggest that not being physically active may be related to poorer physical and cognitive health, with specific concerns surrounding memory. Future research is essential in this area to tease out of other factor that may be contributing to this relationship and to develop new and innovated modalities for older adults to safely exercise.

